# Mathematical and Computational Modeling in Complex Biological Systems

**DOI:** 10.1155/2017/5958321

**Published:** 2017-03-13

**Authors:** Zhiwei Ji, Ke Yan, Wenyang Li, Haigen Hu, Xiaoliang Zhu

**Affiliations:** ^1^School of Information & Electronic Engineering, Zhejiang Gongshang University, 18 Xuezheng Road, Hangzhou 310018, China; ^2^College of Information Engineering, China Jiliang University, 258 Xueyuan Street, Hangzhou 310018, China; ^3^Chongqing Key Laboratory of Oral Diseases and Biomedical Sciences and College of Stomatology, Chongqing Medical University, Chongqing 400016, China; ^4^Institute of Computer Vision, College of Computer Science and Technology, Zhejiang University of Technology, Hangzhou 310023, China

## Abstract

The biological process and molecular functions involved in the cancer progression remain difficult to understand for biologists and clinical doctors. Recent developments in high-throughput technologies urge the systems biology to achieve more precise models for complex diseases. Computational and mathematical models are gradually being used to help us understand the omics data produced by high-throughput experimental techniques. The use of computational models in systems biology allows us to explore the pathogenesis of complex diseases, improve our understanding of the latent molecular mechanisms, and promote treatment strategy optimization and new drug discovery. Currently, it is urgent to bridge the gap between the developments of high-throughput technologies and systemic modeling of the biological process in cancer research. In this review, we firstly studied several typical mathematical modeling approaches of biological systems in different scales and deeply analyzed their characteristics, advantages, applications, and limitations. Next, three potential research directions in systems modeling were summarized. To conclude, this review provides an update of important solutions using computational modeling approaches in systems biology.

## 1. Introduction

In recent years, the area of precision medicine was significantly promoted by the rapid development of next generation sequencing, which implies lower cost and higher throughput [1, 2]. In the meanwhile, high-throughput mass spectrum was widely used to measure the protein expression and posttranslational modification and then generated various kinds of proteomic and metabolic data [3]. In addition, general public databases and platforms, such as GEO, TCGA, and ENCODE, also provide data for analysis and knowledge discovery [4]. Systems biology, which uses multiomic data for deep analyses and predictions, potentially provides insights of the mechanisms of complicated diseases, particularly as various cancers in human [5–7].

At present, people are more interested in the discovery of new drugs for cancer therapy, even though molecular and cell biology had greatly improved our understanding of many diseases in past decades. The essential linkage between basic science and effective treatment was lost, which is the inference and analysis of biological networks [8]. Computational or mathematical modeling of biological systems at multiple scales is an effective way to discover new drugs for cancer therapy in clinic. In the intracellular scale, these networks explain how cells regulate signaling or metabolic pathways to respond the external perturbations or drug treatment [9]. In the intercellular scale, cell-cell communication networks reflect how different cell types communicate through various ligands to promote tumor growth, metastasis, and angiogenesis [10]. In the tissue scale, how these ligands distribute and diffuse in the 3D tumor space was also valuable to be studied [11]. With the advance of high-throughput technology, systems biology developed rapidly; however, the development of mathematical modeling approaches suffers from new biological questions [12].

In this review, we firstly studied several well-established systems modeling approaches of biological networks, such as ordinary differential equations, Petri net, Boolean network, and linear programming. Secondly, we summarized the typical modeling studies for the cell-cell communications (such as tumor-stromal interactions, tumor-immune interactions, and stromal cell lineage process) in the heterogeneous tumor microenvironment, that is, agent-based model. Thirdly, three potential directions of multiscale modeling in systems biology were deeply discussed. We believe that this work can provide a big picture of systemic modeling in systems biology as well as promoting the development of precision medicine in the near future.

## 2. Several Classical Systemic Modeling Approaches

**Figure 1:**
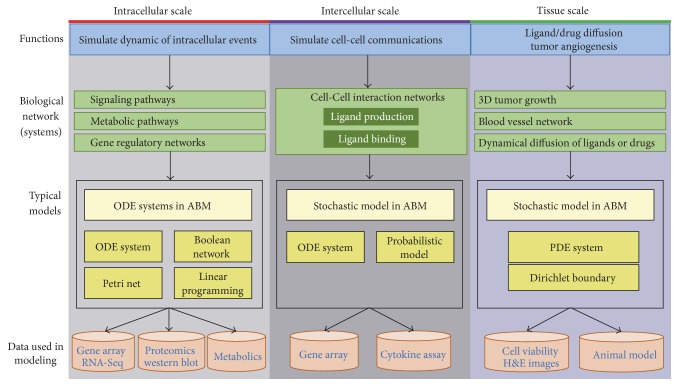
The whole picture of the systemic modeling approaches introduced in this work.

With the development of the high-throughput experiment technologies (such as gene microarray, RNA-seq, mass spectrometry, and metabolic profiles), computational and mathematical modeling of biological processes provides deep insights of the complex cellular systems [13]. Researchers built various computational models to elucidate the complex behaviors of cancers, such as tumor progression, drug resistance, and immune inert. It is well-known that bioinformatics is data-driven [14, 15]. However, systems biology is hypothesis-driven [16, 17], since we often generate a testable hypothesis based on small-scale experimental observations and then construct a systemic model based on this hypothesis to obtain mechanistic insights. In this study, we mainly focus on several classic systemic modeling approaches and their applications in current cancer research. These popular modeling approaches can simulate the dynamic changes of regulatory networks (signaling pathways and metabolic pathways), tumor growth, and its microenvironments, such as ordinary differential equations (ODEs) [10], Boolean network [18], Petri nets [19], linear programming (LP) based model [9, 20], agent-based model [11], and the system biology modeling approach considering genetic variation [21]. We present these models in Figure 1. Although there are many available reverse-engineering [22] algorithms for the inference of gene regulatory networks [23], such as ARACNe [22] and MINDy [24], we omitted them in this review, since they are better suited to be categorized in the field of bioinformatics.

### 2.1. ODE-Based Modeling

With the rapid development of computer performance, ordinary differential equation (ODE) based approaches are widely used for continuous dynamic modeling in complex biological systems [25]. ODE-based methods represent the interactions among various biological molecules (such as protein kinases or metabolites), which reflect the time-varying effects of biological processes [26]. Based on the different biological hypotheses, the current ODE-based methods can be categorized into three types: the* law of mass action* [27, 28],* Hill function* [29], and* Michaelis-Menten Kinetics* [30]. The choice of a specific method depends on the biological questions or the experimental data. Here, we illustrate how to use these kinetic approaches to describe the biochemical reactions.

*Law of Mass Action*. The law of mass states that the reaction rate is proportional to the probability of the collision of the reactants. This probability is also proportional to the concentration of reactants to the power of their molecularity and the number of them entering the specific reaction [31]. For example, a reaction between *A*,  *B*, and *C* can be represented as

(1) $$\begin{array}{c} A+B\overset{K_{ON}}{\underset{K_{OFF}}{⇄}}C \end{array}$$

By the definition of mass action law, we can derive the concentration change over time of the above two reactants (*A* and *B*) and one product (*C*) by the following ODEs:

(2) dAdt=dBdt=KOFFC−KONABdCdt=KONAB−KOFFC

*Hill Function*. In the ODE models of signaling pathways, Hill functions are generally used to represent a protein's activation or inhibition, which are induced by their upstream parental nodes. In biochemistry, the binding from a ligand towards a large molecule can be enhanced if there is also another ligand binding to it, which is called cooperative binding. For each protein involved in the signaling pathways, the dynamic changes of its expression can be described by Hill functions as shown in the following formula:

(3) dydt=∑i=1Mf+xi+∑j=1Nf−xj−y∗dy

(4) f±x=kxyx±nxHx±nx+x±nx,

where *y* is the concentration of activated protein, *x*_*i*_  (*i* = 1,2,…, *M*) is the *i*th protein which activates protein *y*, and *x*_*j*_  (*j* = 1,2,…, *N*) represents the *j*th protein, which inhibits protein *y*. In formula (4), *f*_±_(*x*) is the activating profile (+) or inhibiting profile (−) induced by protein *x*, respectively; *k*_*xy*_ represents activating rate (+) or inhibiting rate (−); *H*_*x*_ is the microscopic dissociation constant and *n*_*x*_ is the Hill coefficient; *d*_*y*_ is the degradation rate of protein *y*.

*Michaelis-Menten Kinetics*. When a signaling reaction is catalyzed by an enzyme (kinase or phosphatase that is not consumed or produced), it may form a temporary complex with substance in the reaction. For such reaction, the Michaelis-Menten Kinetics can be used to describe the reaction rate under the key assumption of quasi-steady-state approximation, while enzyme concentration is much lower than the substrate concentration and the enzyme is not allosteric [11]. The Michaelis-Menten Kinetics is expressed as

(5) $$\begin{array}{c} v=\frac{V_{max}S}{K_{m}+S}, \end{array}$$

where *K*_*m*_ is the Michaelis constant.

Generally, ODE systems are suitable for modeling small-scale networks, since there are many parameters need to be estimated. If the network scale is large, parameter estimation will lead to high computational cost, and the prediction accuracy of model may decrease. Therefore, searching of the optimal parameters for an ODE system is a challenging question. Intelligent algorithms with heuristic search, such as Genetic Algorithm (GA) or Particle Swarm Optimization (PSO), are often used as a heuristic strategy to obtain the parameters in ODE functions [28, 32]. In addition, scatter search potentially find solutions of a higher average quality than GA [33, 34]. Moreover, Stochastic Ranking Evolution Strategy (SRES) is incorporated in some computational strategies for parameter estimation in biological models including signaling pathways and gene regulation networks, such as* SBML-PET* (Systems Biology Markup Language-based Parameter Estimation Tool) [35] and* libSRES* (C library for Stochastic Ranking Evolution Strategy for parameter estimation) [36].

ODE-based approaches have been used in the underlying mechanisms of complicated diseases in* intracellular* and* intercellular* levels. Peng et al. defined a series of ODE-based approaches with the law of mass action, to creatively simulate the intracellular pathways and obtain important biological discoveries [37–39]. For example, they developed an ODE-based modeling approach to comparably assess the inhibition effects of single or combined treatment of drugs on NFKB pathway in multiple myeloma cell and further predict the synergism of drug combinations [38]. Shao et al. proposed ODEs with Hill functions to study the signaling network signatures by integrating both therapeutic and side effects. This model was used to screen 27 kinase inhibitors for optimal treatment concentration [29]. Sun and colleagues designed an ODE-based model with Michaelis-Menten for modeling the antiapoptotic pathways in prostate cancer and illustrated the molecular mechanisms of psychological stress signaling in therapy-resistant cancer [30]. Furthermore, ODEs were also successfully applied to describe the dynamic changes of metabolites in the small-scale metabolic reaction systems [40].

In particular, ODEs have begun to be used to model the cell-cell interactions [10, 32]. For example, Peng and coworkers developed a novel ODE system to understand how the cell-cell interactions regulate multiple myeloma initiating cell fate [32]. The results from this dynamic system may be potentially useful for understanding mechanism of cancer stem cells development. Peng et al. proposed a multiscale multicomponent mathematical model to explore the interactions between prostate tumor and immune microenvironment using ODE-based strategy in both intercellular and intracellular levels [10]. This study highlights a potential therapeutic strategy in effectively managing prostate tumor growth and provides a framework of systems biology approach in studying tumor-related immune mechanism.

Together, all above works indicate that ODE-based models are suitable for modeling the continuous changes of kinetics in small-scale intracellular or intercellular networks [41, 42].

### 2.2. Petri Net-Based Modeling Approaches

**Figure 2:**
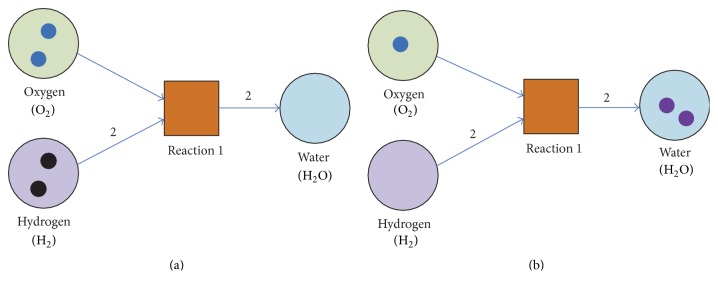
An example of Petri nets (selected from literature [43]). (a) shows the initial marking before firing the enable transition* t*; (b) shows the marking after transition labeled reaction 1 fires.

Petri net (PN), developed by Petri in 1962, was a graphical mathematical modeling tool applicable to a wild range of technical systems [43]. Recently, increasing number of studies are involved in the utility of PNs in systems biology such as modeling of signaling pathways, metabolic pathways, and gene regulatory network [19, 44, 45]. A PN is a directed, weighted bipartite graph consisting of two types of nodes:* places* and* transitions*. As shown in Figure 2, places are represented by circles and transitions are represented by boxes. Through transition firings, the source influences the number of tokens assigned to the target, called* token-count*. A place that has an outgoing arc towards a transition *t* is known as input place of *t*; a place that has an incoming arc from a transition *t* is output place of *t*. Arcs are labeled with weights that represent the minimum tokens required by input places to enable the transition. When a transition fires, it removes a token from each place connected to it by inputting arcs and adds a token to each place connected to it by output arcs. Therefore, a Petri net can be defined as a 5-tuple (*P*, *T*, *F*, *W*, *M*_0_), where *P* = {*p*_1_, *p*_2_,…, *p*_*m*_} is the set of places and *m* is the total number of places, *T* = {*t*_1_, *t*_2_,…, *t*_*n*_} is the set of transitions, the set of arcs *F* = (*P* × *T*)∪(*T* × *P*) and *W* : *F* → *N*,  *N* being the set of natural numbers, is called the weighting function. In order to simulate a dynamic process, a number of tokens are assigned to each place indicating the quantitative property. This assignment of tokens to all the places represents the system states, which is called a marking. The initial marking of a PN is a mapping *M*_0_ : *P* → {0,1, 2,…}. From the initial state *M*_0_, changes in the system are simulated by executing the PN, and a series states can be obtained: (*M*_1_, *M*_2_,…).

For biological molecular network modeling, we usually consider a token to be a unit of weight of a molecule. A place-transition or transition-place connection is made by a weighted arc (directed edge), designating how much of the input places (reactants) are required to produce tokens for the output places (products) in a reaction. A transition can only fire when it is enabled, meaning that each of its input places has at least one token in the current marking. The transition may fire (reaction occurs) afterwards. If transition *t*, when fired on a marking *M*_1_, produces marking *M*_2_, then we write *M*_1_∣*t* > *M*_2_. Obviously, this notation can be extended to represent the effect of firing a series of transitions: *σ* = (*t*_1_, *t*_2_,…, *t*_*j*_).

As a type of dynamic modeling approach, PN was mainly used to simulate not only signaling pathway networks [46, 47] but also gene regulatory networks [48]. In a signaling Petri net, its aim is to predict signal flow through a cell-specific network in experimental conditions [49]. Each place is denoted to the activated signaling protein; and each transition is associated with a unique phosphorylation event.

In fact, PN is a discrete dynamic modeling strategy, which simulates signaling network with multiple states of places. After the topology of a network is determined, PN is suitable to analyze the global property of the system characterized as concurrent, asynchronous, distributed, parallel, nondeterministic, and stochastic. As a novel systemic modeling strategy to describe the biological systems with graphical notation, PN can be used at multiple levels of abstraction and accommodate timing information. Therefore, it forms a language that allows the automatic generation of a specific simulation. However, the obvious disadvantage of PNs is that the conception of PNs is too primitive so that the graphical representation may become too complex for analysis. Hence, developing optimized PN models to reconstruct the specific biological networks is still an important topic.

### 2.3. Boolean Modeling Approaches

In a Boolean network, each node is described with binary states, which are denoted by 1 and 0, corresponding to, for example, activation/inactivation of a protein, respectively. The time variable is considered to be discrete. The future state of a node at each time step is determined by the current states of all its input nodes (parents) through a Boolean function. For each Boolean function *F*, it can be represented as a mapping *B* : {0,1}^*k*^ → {0,1}. This mapping denotes that the outcome of a node was determined by its *k* parent nodes. And the mapping *B* also can be represented as a truth table [13]. These Boolean functions are usually expressed together with the logical operators, including AND, OR, and NOT. Given a Boolean network including *n* nodes, there are *n* Boolean variables (*σ*_1_, *σ*_2_,…, *σ*_*n*_) and *n* Boolean functions (*B*_1_, *B*_2_,…, *B*_*n*_). There are generally two types of strategies for updating the network states: synchronization [50] and asynchronization [51]. In the synchronous pattern, the states of all the nodes are updated simultaneously as shown in the following formula:

(6) $$\begin{array}{c} \sigma_{i}\left(\;t+1\;\right)=B_{i}\left(\;\sigma_{i_{1}}\left(\;t\;\right),\sigma_{i_{2}}\left(\;t\;\right),\ldots ,\sigma_{i_{k_{i}}}\left(\;t\;\right)\;\right). \end{array}$$

The asynchronous pattern can be expressed by the following formula:

(7) $$\begin{array}{c} \sigma_{i}^{*}\left(\;t+1\;\right)=B_{i}\left(\;\sigma_{i_{1}},\sigma_{i_{2}},\ldots ,\sigma_{i_{k_{i}}}\;\right). \end{array}$$

Formula (6) indicates that the state of node *σ*_*i*_ at time point *t* + 1 was determined by the combination of the states of its *k*_*i*_ parent nodes at time point *t*. However, the input variables on the right side of formula (7) might be at different time points.

**Figure 3:**
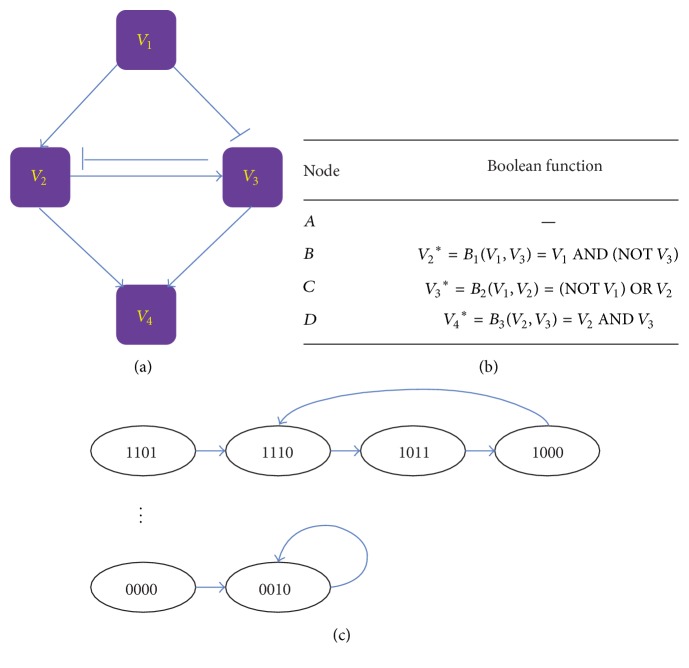
An example of Boolean network. (a) Network topological structure; (b) the definition of Boolean functions; (c) state transition of Boolean network.

The state of a Boolean network at each time step can be expressed as a vector whose elements represent the state of all the nodes at that time step. By updating the states of nodes at each time step, the network states vary over time, which is called “state transition” in BN [9, 52]. In fact, Boolean network modeling is between static network model and continuous network model (such as ODEs), which is especially suitable for large-scale network and is reputed for its high efficacy [9]. An example of Boolean network is shown in Figure 3, to elaborate the above conceptions.  Figure 3(a) shows the topological structure of a Boolean network, which includes four nodes *V*_1_,  *V*_2_,  *V*_3_, and *V*_4_, and their states are defined by three Boolean variables *σ*_1_,  *σ*_2_,  *σ*_3_, and *σ*_4_. Three Boolean functions are listed in Figure 3(b).  Figure 3(c) shows the state transition graph. Given a starting state, the network will eventually converge to a steady state, which is called “attractor” [52]. According to Figure 3, we can easily conclude that the analysis of Boolean network always depends on an assumption: the network structure is determined in advance.

Generally speaking, Boolean dynamic modeling of regulatory network follows three steps: (1) reconstructing the network; (2) identifying Boolean functions from the network topological structure; (3) analyzing the dynamics of the system with or without node perturbations. As a parameter-free model, it works efficiently even for large-scale networks.

During the last decade, researchers carried out many studies of reconstruction of gene regulatory networks using Boolean network modeling [18, 53], which reflects the generic coarse-grained properties of large genetic network. The hypothesis for the Boolean networks as models of gene regulatory networks is that, during regulation of functional states, the cell exhibits switch-link behavior. This hypothesis is important for cells to transfer from one state to another during a complex biological process after the cells received the external stimulations or perturbations [54]. D'haeseleer et al. discussed the way to cluster coexpression profiles to infer large-scale gene regulatory network from high-throughput gene expression assays [55–57]. Moreover, the intrinsic properties of Boolean network, such as stability [58, 59], robustness [60], and fragility [51] for gene regulatory networks were deeply studied, which speeded up the Boolean network development and applications in more areas. In addition, Boolean network modeling approaches have been improved in other fields. Zhao and Ouyang et al. proposed an algorithm for inferring gene regulatory networks by using modified Boolean network from a time series dataset [61–63]. However, Boolean network only provides a very limited quantitative insight in biological systems due to their inherent qualitative nature of state and time. In order to overcome the deterministic rigidity of BNs, probabilistic Boolean network (PBN) was introduced for the modeling of gene regulatory networks [64–67]. Generally, PBNs combine the rule-based modeling of Boolean networks with uncertainty principles as described by Markov chains [68]. Modeling with PBNs provides a quantitative understanding of biological systems, such as interactive effects between genes or average activities of certain genes given by steady-state probabilities [69]. PBNs thus have been widely applied to various biological processes [64, 66, 70, 71].

In recent years, Boolean dynamic modeling begins to be applied to signal transduction networks analysis. Anderson et al. proposed an approach for integrating gene set enrichment methods with Boolean dynamic modeling to reveal the induction of a densely connected network of cellular (TFs) and molecular (ligands) signaling upon influenza virus infection of dendritic cell [72]. Kaderali and colleagues also inferred signaling pathways from gene knockdown data using Boolean networks with probabilistic Boolean threshold functions [73]. PBNs were also applied to study the crosstalk relevancy in a given signaling pathways or simulate the outcome with external perturbations [67, 74].

Since many years ago, Boolean network was widely applied in the studying of network modeling and analysis; however, the key signaling pathways for some diseases or cancers are still unknown, which limits the use of BN in such cases. Although the topological structure of signaling pathways can be determined with the prior knowledge, the specific pathways of some cancer cells are still unclear. Based on above questions, Saez-Rodriguez et al. developed a novel discrete logic model to infer cell-specific pathways [75]. The proposed model represents the topological structure of signaling pathway as Boolean network (two states of nodes). Based on the experimental observations on a part of protein nodes, this model infers an optimal subnetwork from the original generic pathway map as the cell-specific pathways for further predictions. This is the first work to implement the inference and optimization of cell-specific pathways using the conception of Boolean or discrete networks.

### 2.4. Linear Programming Approaches

Linear programming (LP), as an important subject in mathematics, first appeared in the 1950s [76]. Linear programming is the problem of maximizing or minimizing a linear function over a convex polyhedron specified by linear and nonnegativity constraints. Simplistically, linear programming is the optimization of an outcome based on some set of constraints using a linear mathematical model. In general, linear programming models can be categorized into four types: (1) Integer Programming (IP), defining a part of or all variables as integers; (2) Binary Linear Programming (BLP), denoting all variables as binary numbers [9]; (3) Mixed Integer Programming (MIP), constraining that only a part of variables are integer [77]. In addition, nonlinear programming is also very common in the real world, which refers to the mathematical programming which has nonlinear constraints or objective function [78], such as Mixed Integer Quadratic Programming (MIQP) [79].

In recent years, LP-based approaches were applied in the reconstruction of gene regulatory networks [80], metabolic networks [20], and inference of cell-specific signaling network [9, 81, 82]. In the following paragraphs, we will summarize how LP is utilized to model these molecular networks.

**Figure 4:**
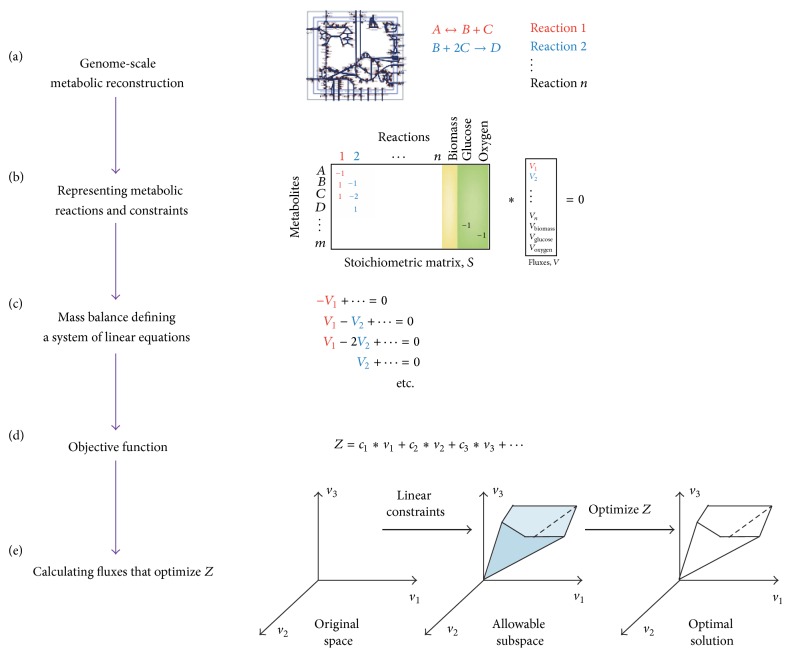
Flux balance analysis for metabolic network reconstruction (selected from literature [20]).

Orth et al. creatively proposed a novel computational approach for the genome-scale metabolic network reconstructions that is called flux balance analysis (FBA) [20]. The metabolic networks contain all known metabolic reactions in an organism and the genes that encode the enzyme of each reaction [83, 84]. The fundamental theory of FBA is that the flowing energy between input and output should be balanced; and FBA makes it possible to predict the growth rate of an organism or the production rate of a biotechnologically important metabolite. FBA-based approaches reconstruct large-scale networks; and LP is an effective strategy to solve this kind of optimization problem [20]. The general work flow of FBA is shown in Figure 4. According to the concept of FBA, a metabolic network reconstruction consists of a list of stoichiometrically balanced biochemical reactions; and each reaction is controlled by an enzyme (Figure 4(a)). The reconstruction is converted into a mathematical model by forming a stoichiometric matrix *S*, in which each row represents a metabolite and each column represents a reaction (Figure 4(b)). At steady state of the network, the flux of each reaction is given by *S* · *V* = 0, which defines a combination of linear equations (Figure 4(c)). By defining an objective function, the linear programming with a set of constraints identifies the solution vector *V* in a subspace (Figures 4(d) and 4(e)). On one hand, FBA provides a way to reconstruct metabolic network in the scale of entire genome. On the other hand, most FBA applications do not consider the thermodynamic realizability. Hoppe was the first to put forward a method by including metabolite concentrations in flux balance analysis [85]. They demonstrated the usefulness of their method for assessing critical concentrations of external metabolites preventing attainment of a metabolic steady state.

Another very important aspect of the LP-based strategies is that those strategies were developed to infer cell-specific signaling pathways with experimental proteomic data [9, 81, 82, 86]. The* rational* of this type of approaches is that the relationships (states) between a child node and its connected parental nodes were defined by a set of constraints. According to the experimental observations of a part of nodes, some redundant edges in the network can be detected and then removed in the process of optimization. The inferred cell-specific signaling network therefore generally becomes a subgraph of the generic pathway map. Mitsos was the first researcher to propose an ILP-based approach for inferring cell-specific signaling pathways with phosphoproteomic data and identify drug effects via pathway alterations [82]. In Mitsos's approach, the protein nodes were represented as Boolean states (“activated” or “inactivated”), which is similar to the Boolean network. The innovation is that the edges were defined by Boolean variables; and the states of connected nodes were constrained by mathematical rules. Therefore, given the observations of a part of nodes in the network, the states of all the nodes and links can be predicted with the defined constraints, and the inconsistent edges are removed from the topological structure of the generic pathways. However, the constraint system developed by Mitsos can only address very simple topological structures of singling pathways; and the generalization of their method is so limited. We proposed an improved Boolean Integer Programming based approach to overcome this limitation and defined four linking patterns of connected proteins for modeling the complex signaling networks [9]. However, Boolean states defined in above approaches have obvious insufficiency for the variations of phosphor-signals under different conditions. Thus, we further proposed a novel discrete modeling strategy (DILP) to represent relative changes of phosphor-proteins with three states (0, −1, and 1, denote “no change,” “downregulation,” and “upregulation”); and the edges are still with binary states. DILP was then applied to infer osteoclasts-mediated myeloma cell-specific pathways under normoxic and hypoxic condition on time series proteomic data and finally revealed that how OC-myeloma interaction in a hypoxic environment affects myeloma cell growth and drug response [81]. In summary, as a type of parameter-free method, LP-based model provides an innovative strategy for modeling large-scale molecular networks.

### 2.5. Agent-Based Model of Biological Systems

An agent-based model (ABM) is another class of computational models for simulating the actions and interactions of autonomous agents with a view of assessing their effects on the system as a whole [87]. In systems biology, ABM is usually used to model tumor growth (drug response) and angiogenesis in the cancer microenvironment. Each cell type in this system is considered as an agent, and the complicated cell-cell communications are achieved through some secreted ligands [88–94]. The implement of ABM is usually based on Markov Chain Monte Carlo. By ABM modeling, we can stimulate the process of stromal cell lineage, tumor growth, and angiogenesis in the cellular and tissue level and the process of signal transduction in the molecular level. Some typical research works about ABM modeling were summarized as follows.

Currently, there are two types of agent-based models: 2D ABM [95] and 3D ABM [96]. Solovyev et al. proposed a two-dimensional agent-based model of ischemia-induced hyperemia and pressure ulcer formation. This model defined a 2D space of pressure ulcer formation and simulated the interactions among skin cells, inflammatory cells, and blood vessels through the cytokines TNF*α* and TGF*β* [95].

**Figure 5:**
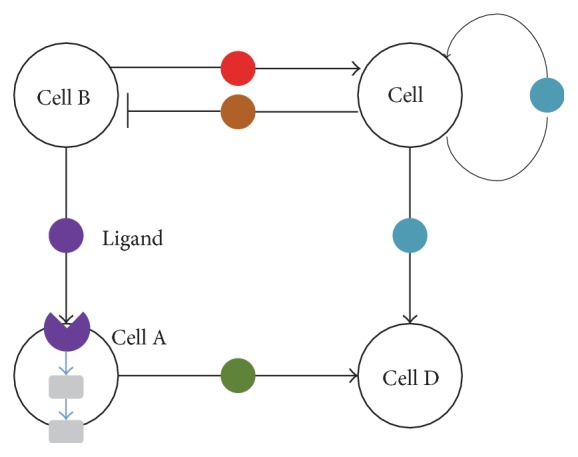
A general framework of agent-based model.

3D ABM models in systems biology were often designed to mimic the dynamic changes of tumor tissues and various interactions with other types of cells (stromal cells, tumor cells, and immune cells) in the heterogeneous microenvironment. Therefore, 3D ABM models potentially integrate events at different spatial and temporal scales. For the spatial scales, the cell behaviors, such as tumor cell migration and invasion and effector cell-induced clearance of target cells, can be simulated in the 3D space. For the temporal scales, short-term intracellular signaling dynamics; medium-term cell division and apoptosis; long-term drug response and tumor growth also can be modeled. Su et al. established an ABM model using the Markov Chain Monte Carlo approach to simulate the effects of SDF1 induced chemophysical communications among MICs and BMSCs on myeloma cell growth and examine whether the biophysical properties of myeloma niches are druggable with two representative drugs [96]. This study provided a typical approach to simulate the process of tumor-stromal cell lineage and intracellular signal transduction with Hill function. Particularly, we further developed a hybrid multiscale agent-based model (HABM) that combines an ODE system and agent-based model [97]. The ODE system was used to model the dynamic changes of intracellular signal transductions; and the ABM is used to model the cell-cell interactions between stromal cells, cancer cells, and immune system. It is the first work to study systemic modeling of tumor growth and immune response within an integrated 3D model (Figure 5). In addition, tumor progression is related to angiogenesis; therefore, it is necessary to integrate vascularization into ABM model to reflect the endothelial cells interaction with cancer cells through some key factors, such as VEGF [98]. Wang et al. proposed an ABM model that integrates the angiogenesis into tumor growth to study the response of melanoma cancer under combined drug treatment [99]. The diffusions of ligands or drugs in the tumor microenvironments are always simulated by PDE [97].

To consider the real situation in tumor microenvironment, multiscale ABM modeling tries to simulate the cell migration in tissue level, cell-cell communication through ligands in intercellular level, and dynamical signal transduction in intracellular level in an integrated system [11, 100]. For example, Sun et al. developed a multiscale ABM model to study cell responses to growth factors released from a 3D biodegradable porous calcium phosphate bone scaffold. Sun's model reconstructed the 3D bone regeneration system and examined the effects of pore size and porosity on bone formation and angiogenesis.

Although current ABM models are able to simulate the tissues, organs, and microenvironment closely enough to the real situation, the variability of model outcomes should be considered. Therefore, replicates are necessary to calculate the models' average performance. Uncertainty analysis should also be used to represent the variability of the model results.

### 2.6. Integrating Genetic Variation to Infer Cancer Networks

Previously, the systemic modeling of intracellular pathway networks depends on an assumption that all cells in a tumor tissue share the same pathways and the data (e.g., western blot and gene array profiles) used in the process of modeling reflects the average expression of molecules. The heterogeneous genetic variations occurred in tumor cell population are not considered in the existing models. How to* integrate the information of genetic variations in the systemic modeling work is now a new topic*. AlQuraishi et al. first proposed a multiscale statistical mechanical framework integrated genomic, binding, and structural data to predict the effects of specific mutations on PPI networks and cancer-related pathways [21]. Based on the concept of Hamiltonian, they modeled how the mutations in SH2 domains induced network alterations and the experimental results validated the proposed model. We believe this interesting topic will attract researchers' wide attention.

## 3. Current Three Hot Directions in Systems Biology

Cancer is a genetic disease driven by mutations in key genes that lead to uncontrolled growth and abnormal cell behavior. However, the fact that tumor is living in a complex heterogeneous microenvironment drives the tumor progression as well as treatment resistances. Understanding the interplay between homeostasis, heterogeneity, and evolution in cancer progression is currently hot topics. Multiscale computational modeling has strong potential to bridge the gap between precision medicine and translational systems biology, in which quantitative metrics and data guide patient care through improved stratification, diagnosis, and therapy.

### 3.1. Dynamics of Cell-Cell Interactions in Tumor Progression

The reciprocal relationship between cancer cells, the host immune system, and the tumor microenvironment evolves during the cancer progression [101]. How these dynamic and unstable interactions prevent or drive tumor initiation and progression is not well understood. At present, some researchers start to focus on predictive and testable hypotheses of how dynamic cell-cell (tumor-tumor [96], tumor-stroma [102], tumor-immune [103], and immune-stroma [104]) communication affects cancer development, and the responses to various therapies. Due to the complex nature of these interactions, mathematical and computational models are ideal tools to elucidate them and make predictions that are profitable for both experiment and novel therapeutic approaches [105–108]. Su et al. proposed an agent-based model to mimic the multiple myeloma cancer cell lineage process and the interactions between myeloma initial cell (MIC) and bone marrow stromal cells [96]. The computational model simulated the myeloma progression and drug resistance driven by various cell-cell communications. Similarly, to understand the mechanism of multiple cell-cell interactions involved in circulating tumor cell adhesion, Uppal et al. used an ABM of Early Metastasis (ABMEM) to dynamically represent the hypotheses of essential steps involved in circulating tumor cell adhesion and interaction with other circulating cells, examine their functional constraints, and predict effects of inhibiting mechanisms. The results show that the ABMEM successfully captures the essential interactions of the whole process and allows in silico iterative characterization and invalidation of proposed hypotheses regarding this process in conjunction with in vitro and in vivo models [109]. Furthermore, both the innate and adaptive immune response have been demonstrated to induce tumor cell death [110, 111]. Conversely, cancers are assumed that have developed adaptive strategies to evade immune attack. However, systemic modeling of tumor formation, growth (development and progression), and immune functions (including macrophage, CTL, and Treg) within an integrated computational system was still rarely studied. Therefore, modeling the interactions of cancer cells, stromal cells, and immune cells in its microenvironment may potentially improve our understanding of tumor growth, immune tolerance, and drug resistance [97].

### 3.2. Systems-Level Analyses of the Role of the Heterogeneity and the Microenvironment

Usually, researchers consider a tumor to be a heterogeneous population containing potentially many distinct cellular phenotypes (variations of cell-specific traits such as cell-cell adhesion, migration speed, and proliferation rate); and, via proliferation, each cell had a small chance to mutate to one of these phenotypes in a random manner. A number of modeling strategies have suggested that the local microscopic heterogeneity can vary wildly in tumor, such as blood vessel, cellular density, and metabolism [112, 113]. In another aspect, genetic heterogeneity (e.g., mutation) within a tumor continues to be of great interest to the cancer community [114]. A major question in biology is how to connect genotype with phenotype. We believe that, only through the integration of computational models with careful experimentation, the gene-centric and microenvironment-centric views of cancer progression can be bridged.

Furthermore, tumor microenvironment (mE) is temporally and spatially heterogeneous due to the variations in blood flow, resulting in local fluctuations of nutrients (e.g., O_2_), growth factors [115], extracellular matrix, and other cellular populations [116]. The mE is considered to play a crucial role in driving the evolution of aggressive tumor phenotypes; however, the problem of how the mE modulates this heterogeneous and drives the behavior of the tumor cell population is still far away from being fully understandable by researchers [116]. Picco et al. developed a 2D hybrid discrete-continuum cellular model to investigate the role of environmental context in the expression of stem-like cell properties through in silico simulation of ductal carcinoma [101]. They demonstrated that variations in environmental niche can produce cancers independent of genetic changes in the resident cells. Also, Ji et al. investigated the effects of oxygen heterogeneous distribution in bone marrow on myeloma progression via osteoclast-myeloma cell interactions and proposed an LP-based model to infer OC-mediated myeloma cell-specific signaling pathways under hypoxia and normoxia [81]. The above modeling studies guide us to realize the therapies targeting the mE which may offer an alternative cancer prevention strategy.

More recently, quantitative measurement technologies have facilitated the collection of chemical, molecular, structural, interactome, and localization data within and across cell populations in the tumor microenvironment. The systems modeling and analyses using multiomic data are potentially to predict cell behaviors and translate important information across space and time.

### 3.3. Systems Biology Aided Clinical Trial Design

Precision medicine requires integrating patient-specific characteristics into knowledge gained preclinical studies, such as differences in multicellular or multiclonal drug response, staggered temporal dosing schedules, and dynamic prediction of effective combination therapies. Systems biology synthesizes data obtained from individual reductionist perspectives, focuses on construction of integrated, holistic models of determinants of biological responses, and offers an exciting opportunity by which to identify potential therapeutic targets [117]. Currently, methylation marks, transcript abundance, and miRNA profiles are integrated with SNP results from GWAS to better explain the genome complexity as it may relate to pathophenotype and drug treatment response. It is a good way to take clinically relevant cells from clinical trial participants, treat those cultured cells with the relevant drug from the clinical trial, and determine transcript abundance in the cell as a function of drug treatment response. The data can be further integrated with GWAS data from the trial to perform a regression of SNP on transcript abundance in order to identify regulatory variants that can be utilized for pathway modeling or network analysis. More recently, using a systems biology strategy, Laaksonen et al. demonstrated the treatment effects of high dose simvastatin on nonhepatic tissues as well as a different profile of the effects of atorvastatin on such tissues. They described that the further understanding of the impact of their lipidomic findings has the potential to lead to* individualized drug and dose selection* [118]. Another example of the potential benefits of a systemic modeling approach is drug combination therapies [10]. Expansion of the catalog of drug interactions using a systemic modeling approach, incorporating pharmacogenomics and computational biology, has the potential for optimizing pharmacotherapeutic schemes in the future.

Recent developments in molecular analysis and bioinformatics make targeted treatment feasible; however, more efficient, multifaceted clinical trial designs are still needed. The studies on the utilization of systems biology approaches in clinic may help us to refine experimental measurements and improve decision-making about therapies for clinical trial planning and ultimately personalized therapy. Artemov et al. presented a novel computational model for predicting an optimal personalized treatment for cancer patients based on high-throughput gene expression signature of the individual tumor samples. The effectiveness of this model was validated by using clinical trials data [119]. Kim et al. proposed an ODE-based computational framework to implement virtual phase *i* trials in cancer, using an experimentally calibrated mathematical model of melanoma combination therapy, which can readily capture observed heterogeneous clinical outcomes and be used to optimize clinical trial design [120]. Lawler et al. suggested potential solutions in precision medicine for clinical trial design that balanced the cost and value, to deliver cost-effective cancer care [121]. The benefits of Lawler's approach can be transferred directly to the patients [121].

## 4. Discussion

The development of cancer study is a complex, multiscale biological process, in which genetic mutations occur at a subcellular level and manifest themselves as functional changes at the intracellular, intercellular, and tissue scale. Significant developments of integrating mathematical modeling approaches with experimental data provide deep insight of the mechanisms that provoke cancer initiation, progression, and drug resistance. Although various computational models have been developed in cancer research studies, the current challenge is that the knowledge about the mechanistic details of many biological systems is still rare. On the other hand, the real biological systems are much more complicated so that current established mathematical approaches are insufficient to describe all the details in these systems.

In this review, we firstly provide a methodology overview of mathematical and computational modeling in systems biology which was well studied to elucidate how complex behaviors of biological systems are. This category of approaches includes ordinary differential equations (ODEs), Petri net (PN), Boolean network (BN), Integer Linear Programming (ILP) for intracellular network modeling, and agent-based model (ABM) for cell-cell communication in intercellular level. ODE systems are well-suited to describe continuous processes that can be approximated as well-mixed system. ODE-based modeling is often applied to small-scale intracellular network or cell-cell interaction network in systems biology. Boolean network, a typical discrete modeling approach, uses well-defined connectivity information to study the global and dynamical properties of network. Boolean modeling of biological network realizes the state transition of the computational system among discrete time points by defining the states of nodes and edges as binary variables [122]. Petri net, as a discrete dynamic model, is widely used in biological network modeling. PN stimulates the dynamic changes of kinase in signaling pathways as ODEs and represents the state transition between discrete time points as BN. The linear programming based approach is an innovative strategy to model large-scale molecular networks [81]. LP-based method is parameter-free, works efficiently in the optimization of networks, and can be combined with ODEs to develop a two-stage hybrid model for signaling pathway reconstruction. The LP-based method was first used to simplify the generic pathway map to obtain cell-specific pathways. Then, the ODE-based method was applied on the inferred cell-specific network to analyze the dynamic changes of each kinase. Finally, the agent-based model and its usage in biological systems are introduced [100]. Agent-based simulations monitor the actions of a large number of simple agents, in order to observe their aggregated behavior. ABM is not only used to model 2D computational systems [95] (such as skin injury and inflammation) but also used to model 3D tumor growth with its microenvironment [96, 100].

## 5. Parameter Estimation, Sensitivity, and Uncertainty Analyses

### 5.1. Parameter Estimation

**Figure 6:**
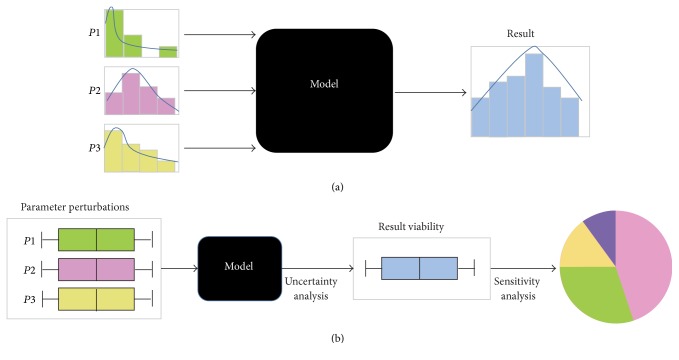
Uncertainty and sensitivity analyses of model output. (a) The baseline of the model; (b) the framework of uncertainty and sensitivity analysis. Based on the changes of each parameter within a range, UA is firstly used to analyze the viability of model results. And then SA is used to identify which parameters are responsible for the result viability.

Most mathematical models in systems biology face three troubles: highly nonlinear models, a large number of parameters for approximation, and the scarce information content of the available experimental data. Hence, there is a demand for global optimization methods that are capable of estimating the parameters efficiently (Figure 6(a)).

ODE is widely used to model signaling pathways and metabolic pathways [29, 123]. The parameters in ODE systems were often optimized by heuristic search algorithms, such as Genetic Algorithm (GA) and Particle Swarm Optimization (PSO) [39, 99]. The classic algorithm of GA or PSO usually plunges into local optima; therefore, some advanced strategies about the global search of parameters in large-scale optimization are required, such as an enhanced* scatter search* algorithm for parameter estimation in large-scale systems biology models [124, 125]. Moreover,* Stochastic Ranking Evolution Strategy* (SRES) is incorporated into some computational tools for parameter estimation jobs [36, 126]. For example, libSRES [36] is a free C library for Stochastic Ranking Evolution Strategy for parameter estimation, which is suitable to use under opens source environment.

However, ABM is a computational model for the stochastic process simulating behaviors and interactions between autonomous agents, where the outcome might be fluctuate even though the parameters were fixed. Generally, parameter tuning is a common way to determine the parameters in ABM, such as Su et al.'s work [96]. We suggest that the researchers may manually determine the parameters in ABM model by tuning and ensure that the simulation outcomes are close to experiment data. For each condition, it is necessary to implement ABM model over hundred times, and the dynamic changes of each cell population can be represented by averaging the results of all the times. By using this strategy, the predicted results of ABM model are reliable and stable.

### 5.2. Sensitivity Analysis

Sensitivity analysis (SA) of parameters provides valuable insights of the parameters that are responsible for the variability of model outputs (see Figure 6(b)). Local and global sensitivity analysis approaches are commonly applied in systems biology [127]. Taking the ODE system as an example, some local sensitivity analyses often increase or decrease only one parameter with a small range at a time to learn the impact of small perturbations on the model outputs [29, 38]. On the other hand, global sensitivity analysis is used to investigate the effects of simultaneous parameter variations over large but finite ranges. It also explores the effects of interactions between parameters [128, 129]. The selection of proper sensitivity analysis approaches depends on the specific biological models and the experimental data.

### 5.3. Uncertainty Analysis

Uncertainty analysis (UA) evaluates how the variability of parameters propagates through the model and affects the output values [130]. Different from SA, which evaluates how parameter variability contributes to model output, the objective of UA is to quantify the distribution of results given uncertain parameters (see Figure 6(b)). In the process of UA, it requires multiple models to run, where parameter values are randomly chosen from their respective distributions. The selection of the sampling methods used to perform UA is essential. The quasi-random sampling that generates samples more uniformly over the entire parameter space is widely used [131]. In UA, the mean represents the central tendency of the stochastic process; and the variance summarizes the variability of the model output.

Secondly, we summarized several hot topics of systems biology in cancer research. Modeling the dynamics of cell-cell interactions in the process of tumor progression provides significant insight into the mechanisms of cancer development in the complicated microenvironment. System-level analyses of the role of the heterogeneity discussed the differences between patients with the same type of cancer, the different cancers for the same patient, the behaviors of various cell types among the tumor tissue, and the different genomic changes among the same type of cells. Obviously, there are still many complicated biological processes and phenomenon that are not explored or understood by human.

In summary, in order to simulate and represent more complicated biological systems, the current modeling approaches introduced in this review are still limited. Another limitation is that we did not further discuss the studies of miRNA-mRNA interactions, although there are some systems biology approaches reported in the literature, which are related to functional genomics based analysis [132]. We expected that, in the near future, the effects of genomic information (e.g., mutation and alternative splicing) on the cell states or cell behaviors can be well modeled. Furthermore, the techniques in computer science and mathematics may provide some new theoretical models for systems biology.
